# A Comprehensive Review on the Impacts of Smoking on the Health of an Individual

**DOI:** 10.7759/cureus.46532

**Published:** 2023-10-05

**Authors:** Jerin Varghese, Pramita Muntode Gharde

**Affiliations:** 1 Community Medicine, Jawaharlal Nehru Medical College, Datta Meghe Institute of Higher Education and Research, Wardha, IND

**Keywords:** risk-factors, negative impact, various systems of human body, harmful effects, cigarette smoking

## Abstract

Long-term smoking for several years has been known to cause severe ailments in humans from the beginning. Even after knowing that this dangerous addiction is a life-threatening deal, still, ironically, the prevalence of smoking is more or less not getting reduced to a desirable extent. Those who smoke are becoming miserable because of their habit of smoking. Still, on the other hand, due to passive smoking, many more innocent lives are also adversely affected for no fault. This aspect of smoking, i.e., passive or second-hand smoking, is a fearful complication of smoking which is seldom seen with other modes of addiction. Time and again, numerous researches have highlighted the adverse effects of smoking on the human body and the interference it does bring in one's life. Smoking contributes to the deterioration of many preexisting ailments and depletes many valuable aspects of the human body. Smoking thus has a devastating effect on almost all of the tissues of our body and thus exerts its effect on nearly all the major organs. This review article is made by analysing various findings from many researches conducted across the globe by having a thorough search of Pubmed database, which in turn is the main methodology of the article. This review article aims to provide a simple and subtle understanding of the ill effects of smoking on the human body by serving the readers with a readymade platter of comprehensive knowledge about smoking coupled with efforts to eliminate the associated myths.

## Introduction and background

Since the beginning of the human race, men have abused various smokes to get euphorbia. Depending on the availability of the smokable stuff, the content being rolled and its effects vary. Numerous types of smoke-producing products are available in the market [1]. Almost all of them, irrespective of the design of the smoke-propelling device, are harmful to the human body. Even after knowing that smoking is a significant cause of several ailments in the community, the prevalence of smoking is ironically increasing daily [2]. The health impacts of smoking have been put forward in the community with the help of various campaigns and other means in simple language [3]. Still, the effect of these campaigns on the education of smoke-related health issues is only short term and in the long run, is not very helpful in making the users of smoke-related products abstain from them in large numbers [4].

Unlike other addictions, one of the most dangerous and feared adverse effects of smoking is that those who smoke are ruining their own life and becoming a risky threat to many more innocent lives around them by paving the way for them to their graves. Passive smoking is a dreaded complication of smoking, and people who have nothing to do with it suffer because of those around them who smoke [5]. Even though smoking is prohibited in public places, the extent to which a smoker pays attention to it is seldom impressive and respectful [6-11]. In families where some member of the family does smoke, other members seldom are spared from the ill effects of passive smoking [12,13]. Strict rules and regulations need to be put forward, and in a way, making sure it is imposed correctly and promptly would solve the issue of passive smoking considerably. Smoking areas exist in many airports, offices, etc., where a person can smoke without letting that smoke reach a non-smoker, and this is a reality already; better strategies like these are needed to prevent passive smoking in our community.

Those who smoke and drink are considered to have more severe health ailments. Drinking and smoking increases the intensity of harm a body needs to bear and is a matter of great concern. The easy availability of smoke-related products near areas where drinks are available is a primary culprit behind this dangerous deed. Enforcements must be planned and carried out successfully to help smokers abstain from this fatal mixture of addictions. Those who smoke need proper care and counselling, and time and over, it has been evident that many smokers benefited and gave up their habit of smoking after such eventful counselling sessions and guidance [14]. The prevalence of such activities needs to be increased and made available to the general public to ensure it is not limited only to the wealthy and affluent class. Media does play a crucial role in the promotion of smoking. Seeing a favorite actor smoking does impact their fans from adopting similar actions [15,16]. Over the past couple of years, the narration of women being smokers has been portrayed in various movies and series, making many female viewers deviate towards smoking. Advertisements of smoke-related products are another way people get influenced to smoke. Although regulations are imposed in this respect on commercial ads, there are many surrogate advertisements in our community where the product is indirectly promoted and advertised by renowned celebrities, sports personalities, social influencers, stars, etc. [17,18].

Undoubtedly, smoking is a major risk factor for many respiratory ailments but the adverse effects of smoking aren't limited to just the respiratory system. The false notion among the community that smoking causes only respiratory-related ailments had prevailed in our community a couple of decades prior. However, as newer research surfaced, this belief got a shattering setback. Today, smoking is considered hazardous to almost all the varied systems of our body, and no doubt further research would add more ill effects of tobacco to the already proven list. Smoking acts as a precipitating factor in the causation of various diseases. Making more and more people aware of the impact of smoking would probably decrease the number of smokers. Mass campaigns that are effective, acceptable and understandable to the general public are needed to save the lives of those addicted to this fatal smoking habit [3,19]. A comprehensive strategy to make the person know the effects of smoking in their personalized life would be a better idea. Many myths revolve around smoking, which needs to be corrected [20]. Considering one form of smoking over another by believing that the latter is better and harmless is common among smokers. There is a constant struggle between the cigarette companies to bring safer cigarettes as it is a worse fact that this fatal habit has destroyed many lives, and the saga is still continuing. Many such products, which look like cigarettes (for example, herbal cigarettes, ayurvedic cigarettes, etc.) are also available in the market, urging smokers to use them safely without any significant health concerns [21,22]. More or less, these products have been a failure at even the ground level as these products were not able to rise to the standard demands of a smoker and thus turned out to be an unacceptable idea for those who smoke. Moreover, a massive lot of anti-nicotine products like anti-nicotine gums, medications, etc., are available for smokers to aid them in getting rid of nicotinic cigarettes, but this is made handy by only a few smokers who are determined to give up smoking; the majority do use such products for a while and then gradually get back to their initial states. Similar misconceptions are frequent in our community, which must be addressed appropriately. Unless and until the layperson is not made aware of the facts, the intensity of damage caused by misinformation will keep increasing.

## Review

Search methodology

**Figure 1:**
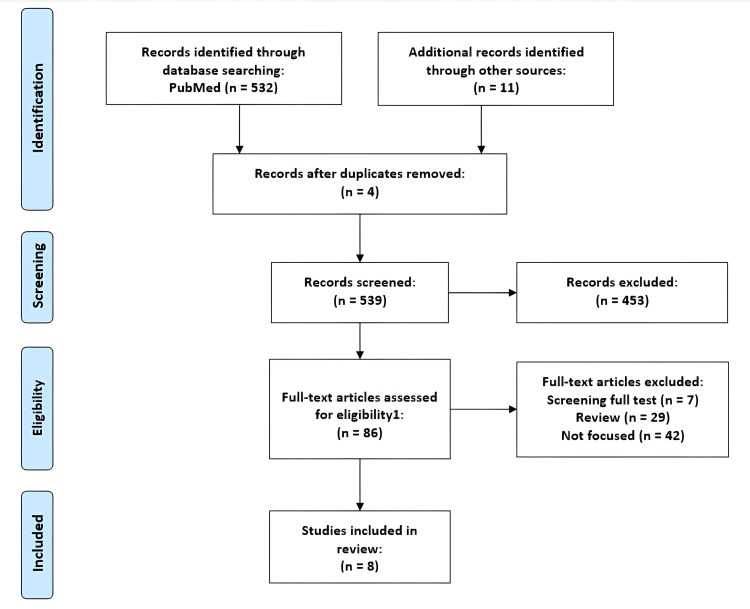
PRISMA flow diagram of the review process

This review article was made after careful assessment and evaluation of the various researches conducted across the globe on the impacts of smoking on the health of an individual. The primary database used for the search is PubMed along with certain researches obtained from other databases and sources. The duration of the publication of articles considered for the purpose of review is within the past 25 years. Those articles which couldn't put forth a definite and precise conclusion and those whose results were found to be doubtful are excluded. A PRISMA flow diagram of the review indicating the screening process is summarized in Figure 1 below.

Effect of smoking on the respiratory system

**Figure 2:**
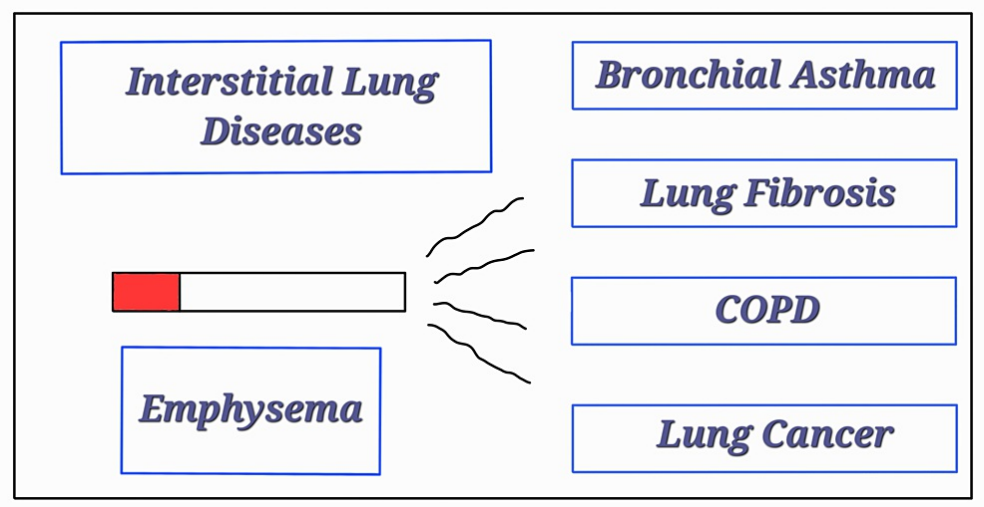
A schematic representation of the impacts of smoking on the respiratory system of an individual COPD = Chronic Obstructive Pulmonary Disease

The various aspects of smoking-related issues concerning the respiratory system are well known to humankind. When we say ill effects of smoking, the first image that runs to our mind is damaged lungs. Undoubtedly, smoking does have a profound impact on our respiratory system. The major respiratory illness associated with smoking includes bronchial asthma, Chronic Obstructive Pulmonary Disease (COPD), emphysema, interstitial lung diseases, lung fibrosis, lung cancer, etc. [23], which is schematically represented in Figure 2 below. Smoking is a crucial factor in the causation and worsening of many respiratory illnesses. It is a common scenario where smoking is portrayed to be directly linked with the respiratory system in many anti-smoke campaigns to educate the general public regarding the impacts of smoking. A large number of acute eosinophilic pneumonia cases do have smoking as a precipitating factor [24]. Almost all of the varied types of pneumonia get worsened and are affected by the habit of smoking. Smoke is a precipitating factor and thus could easily be linked to various pulmonary ailments. Smoking leads to the proliferation of a wide variety of cells. Different hypothesis has popped up over time regarding the proliferation of cells in individuals who smoke, and many of these hypotheses have been scientifically proven to be correct through research. The proliferation of Langerhans' cells is an essential entity in this respect. To a great extent, the proliferation of Langerhans' cells is related to cigarette smoking, thereby leading to Pulmonary Langerhans' cell histiocytosis [25]. More or less, every smoker harms one or the other part of their bronchial architecture due to smoking. Bronchiolitis, an inflammatory bronchial reaction, is often found in those who smoke. It won't be wrong to say that almost all smokers have respiratory bronchiolitis [26]. There has been a firm relationship between smoking and respiratory bronchiolitis-associated interstitial lung disease (RB-ILD) and desquamative interstitial pneumonia (DIP) [27]. The relationship of smoking with respiratory bronchiolitis-associated interstitial lung disease (RB-ILD) and desquamative interstitial pneumonia (DIP) is being further explored to aid in framing effective management strategies to compact these ailments. Idiopathic Pulmonary Fibrosis (IPF) exact cause is not yet known and is suggestive by its name, but cigarette smoking is a significant risk factor for Idiopathic Pulmonary Fibrosis [28]. The correlation between smoking and Idiopathic Pulmonary Fibrosis (IPF) is profound. Emphysema and pulmonary fibrosis are seen in patients with Interstitial Lung Diseases (ILD) who continue to smoke [29]. In fact, smoking is attributed to be a key player in working the cases of Interstitial Lung Diseases (ILD) to such an extent that irreversible changes are brought about in the lungs of the patients suffering from the ailment. Smoking interferes with the homeostasis of many elements in our body, be it macroelements or microelements. The impact of smoking is more dreaded in the homeostasis of microelements in particular, one of the most significant examples being the influence of smoking on iron homeostasis. Iron homeostasis of the cells gets disrupted when exposed to cigarette smoke particles, which ultimately is assumed to lead to the development of non-neoplastic injury of the lungs [30]. Even e-cigarettes interfere with the respiratory health of adolescents with asthma [31], and thus are no better alternative. Lung perfusion in Magnetic Resonance Imaging (MRI) studies was found to be increased after using electronic nicotine delivery systems, while it decreased exposure to tobacco smoke [32]. The prognosis of respiratory illness is worsened by the habit of smoking to a great extent. Whatever the source of smoke, almost all the varied smoke is harmful to the proper functioning of our alveolar complex. The deposition of toxins and tar in the lungs is a dreaded complication of smoking, leading to irreversible damage to the typical architecture over prolonged years. The incomplete combustion of smoke-producing substances adds to the terror, and many researchers have highlighted that these substances that are not entirely burnt cause more adverse pulmonary ailments when compared with those that burn out completely.

Effect of smoking on the cardiovascular system (CVS)

Smoking causes various cardiovascular ailments in humans and is a leading risk factor in the causation of cardiovascular diseases worldwide; it is also a preventable cause of mortality in individuals with cardiovascular diseases [33]. Abstinence from smoking is often advised after almost all of the cardiovascular events, whether it be a major or minor one, which showcases the influence of smoking on a person's cardiovascular health. Various studies have shown that those who had abstained from smoking benefited from the same, as there was a notably better prognosis of cardiovascular ailments in these individuals compared to those who continued smoking. It is a clearly evident and scientifically proven fact that the prognosis of cardiovascular events is adversely affected when a patient with cardiovascular ailments smokes. Almost all of the cigarettes in the market contain nicotine in some or other amount. Nicotine is a primary cigarette component, accelerating atherogenesis, thereby leading to coronary artery heart disease [34]. Abolishing the false belief that hookah smoking is far less harmful than conventional cigarettes is necessary. Because of hookah's overlapping toxicants and chemical profile, its harmful effects on the cardiovascular system are comparable to regular cigarettes [35]. The constituents of hookah smoke, like carbon monoxide, oxidants, phenols, nicotine, heavy metals, etc., are proven to cause serious cardiovascular issues in those who smoke them [36]. Hence, considering hookah as an alternative to cigarette smoking is no good. Waterpipe smoking, too, has adverse cardiovascular effects, which at times are comparable to the smoking of regular cigarettes [37] and thus has to be avoided. Cigarette smoking causes dysfunctioning of the cardiovascular system by many mechanisms, one of the most common and notable being the mechanism involving an increment of oxidative stress [38]. An increase in oxidative stress is bound to damage the structures of the cardiovascular system. Passive smokers also suffer from cardiovascular ailments because of the smoking habits of the people near them. In second-hand smoking, vascular inflammation is promoted by increased oxidative stress [39]. The effects of second-hand smoke on the cardiovascular system are fast and substantially comparable to those caused by primary smoking [40]. The levels of cardiovascular disease risk biomarkers like homocysteine and fibrinogen are disproportionately increased in passive smokers, which points to the fact that passive smokers suffer from cardiovascular disease risk [41]. The elevated levels of C-reactive proteins in second-hand smokers indicate the long-term cardiovascular risk in these individuals [42]. Thus, from the frame of cardiovascular health, both smokers, i.e., the primary and the second-hand smokers, are affected similarly in many respects. Serum levels of cotinine were on the higher side in second-hand smokers compared to those without exposure to smoke, which alarms that second-hand smokers are at a greater risk [43] when compared to the real culprits. The myocardium and the artery vessels are adversely affected by the smoke, and thus, this leads to worsening cardiovascular health [44]. Many radiological findings reinforced and validated that smoking causes remarkable changes in an individual's cardiovascular system. B-mode ultrasound measurement of carotid artery intimal-medial thickness showed increased smoking led to increased initial-medial thickness [45]. Specific, irreversible changes are brought about by long-term smoking in the progression of atherosclerosis [46]. The prognosis of hypertension is badly worsened as a result of smoking. Because of an accelerated rate of atherosclerosis in hypertensive smokers, hypertension usually progresses to renovascular and malignant hypertension, which are severe forms of hypertension [47].

Effect of smoking on the reproductive system

Smoking adversely affects men's and women's reproductive systems. The fetus, too, suffers because of the smoking habits of a pregnant woman. The influence of smoke on the intrauterine life of a fetus is well known, and smoking is contraindicated during pregnancy. Women who smoke should stop smoking at any cost to have a healthy baby. It is a heartbreaking reality that a fetus seldom gets spared from being a victim of various ailments because of the act of smoking by their pregnant mother. Considering the multiple aspects of smoking-related issues linked with the reproductive capability of an individual, one could say that smoking is a great curse for a healthy reproductive life. Smoking causes testosterone imbalance in men along with the causation of erectile dysfunction, muscle weakness, etc. In women, undesirable hormonal changes occur due to long-term cigarette smoking, thereby interfering with proper reproductive functionality. Especially during gestation, the continuation of smoking is undoubtedly a contrary factor to the birth of a healthy baby. Smoking is linked with erectile dysfunction, thereby contributing to infertility in males. The normal physiology of the nitric oxide signal transduction pathway is altered because of cigarette smoking, which is thought to be the main contributing factor in the causation of erectile dysfunction in individuals who smoke [48]. Erectile dysfunction is often related to endothelial diseases, and smoking is a significant risk factor in developing endothelial diseases; thus, smoking does affect erectile function by its effect on the causation of endothelial diseases [49]. Documentation supports the fact that there is a dose-response relationship between the risk of erectile dysfunction and cigarette smoking [50]. Smoking, to any degree, is harmful to a man's reproductive health, and thus, one must abstain from it. Smoking has a severe negative impact on the hormonal balance of an individual, which, when coupled with the effects of smoking on other organs linked with reproduction, leads to complex fertility issues [51]. Although cigarette smoking doesn't have a massive impact on the testosterone fraction that is available biologically, it brings about changes in the sex hormone binding globulin (SHBG), thereby having its say in the free and total levels of testosterone in a man who smokes [52]. SHBG is an essential factor that determines the reproductive fitness of an individual, and thus, smoking, with its harmful effect on the SHBG, definitely worsens the reproductive health of the concerned. Reduced femur length and head size after the first trimester are frequently found in women who smoke during pregnancy [53]. Reduced fetal measurements are seldom seen in the first trimester, but it is common in the second and third trimesters in cases of maternal smoking [54]. This doesn't mean it is safe to smoke in the first trimester. Children born to women who used to smoke during their pregnancy have been found to have lower birth weights [55]. Intrauterine growth retardation is considered to be the attributable factor causing the low birth weight of newborns in maternal smokers [56]. Significant intrauterine retardation of growth has been found in the babies born to mothers who smoke. Maternal smoking has been reported to cause developmental delay issues in children, some of which are almost irreversible and fatal. Infants exposed to smoke are more prone to long-term adverse health impacts than those not exposed. Also, a pregnant woman should take special care to keep herself and her growing fetus safe from second-hand smoke, which has similar adverse effects.

Other effects of smoking

Smoking does cause relevant ill effects on many of the organs of our body, which is seldom understood and given very little importance by the general public due to the lack of knowledge and mere ignorance. These changes that smoking brings to our body may sound less harmful at first, but they may even be permanently irreversible when they progress to an advanced level. Smoking reduces the generalized immune status of an individual, and smokers are more prone to ailments than those who don't smoke. Smokers get infected by pathogens more readily than non-smokers. Smoking harms the oral immunity of an individual. Smoking reduces the host's oral immunity against Candida albicans and thus makes the individual susceptible to oral infections involving Candida [57]. The natural oral and nasopharyngeal bacterial flora gets disturbed due to smoking, thereby leading to the colonization of these sites by potentially pathogenic organisms [58]. Smokers have lesser aerobic and anaerobic microorganisms with interfering capacity than non-smokers, threatening the normal oral flora [59]. The disturbed composition of the flora gets reversed on cessation of smoking, highlighting that smoking is the primary factor responsible for disturbed oral flora [60]. In cases of chain smokers, it becomes more or less impossible for the normal oral flora to get reestablished. The reduction in smoking is estimated to reduce the probability of the development of Alzheimer's disease [61], thereby having its say in a condition that has a profound impact on an individual's life. Otoacoustic Emissions (OAE) are generally reduced in smokers compared to non-smokers [62]. Smoking cessation leads to a reduction in the incidence of periodontitis and is also helpful in the better prognosis of those undergoing treatment [63]. Smoking is a massive hurdle in achieving and maintaining desirable oral health. Smoking interferes with the skeletal system of our body and is proven to be a crucial factor in the prognosis of many orthopaedic conditions. Smoking causes delayed union of fractures, thereby leading to a more extended recovery period and poorer prognosis [64]. Disorders of the lumbar disc and the metabolism of bones are affected adversely by smoking [65]. Post-operative infection rates steadily increase when the patient who underwent surgery is a smoker [66]. Smoking plays a crucial role in cancers of other organs, too, other than the lungs. Smoking is considered a significant risk factor in the causation of neoplastic changes in most of the malignancies found in human beings in one way or another. In patients with glaucoma, optic nerve vessel density is found to be reduced in association with the intensity of smoking [67]. Smoking leads to dryness of the eyes as it interferes with the normal lubrication of the eyes by causing damage to the meibomian glands of our eyes [68]. The various researches reviewed in order to formulate this review have been summarized in Table 1.

**Table 1:** Summary of the revised articles

SERIAL NUMBER	TITLE OF STUDY	IMPLICATIONS	AUTHOR, YEAR [CITATION]
1	Desquamative Interstitial Pneumonia and Respiratory Bronchiolitis-Associated Interstitial Lung Disease	In most patients, DIP and RB-ILD are related to smoking.	Ryu JH et al., 2005 [27]
2	Cigarette Smoke Particle-Induced Lung Injury and Iron Homeostasis	Cigarette smoke exposure produces a functional iron deficiency following complexation of host metal to reactive groups at the CSP surface.	Ghio AJ et al., 2022 [30]
3	MRI Shows Lung Perfusion Changes after Vaping and Smoking	MRI showed decreased perfusion of the lung after tobacco smoke exposure and an increased perfusion of the lung after the use of electronic nicotine delivery systems.	Nyilas S et al., 2022 [32]
4	Cardiovascular toxicity of nicotine: Implications for electronic cigarette use	Nicotine use leads to accelerated atherogenesis and contributes to acute cardiovascular events in smokers.	Benowitz NL et al., 2016 [34]
5	The pathophysiology of cigarette smoking and cardiovascular disease: An update	Exposure to cigarette smoke is an important cause of cardiovascular morbidity and mortality.	Ambrose JA et al., 2004 [38]
6	Active and passive smoking are associated with increased carotid wall thickness. The Atherosclerosis Risk in Communities Study	There is a strong relationship between active smoking and carotid artery IMT and exposure to passive smoking is related to greater IMT.	Howard G et al., 1994 [45]
7	Smoking and erectile dysfunction: evidence-based analysis	Evidence available as of now on the association of smoking with erectile dysfunction is not complete as association linking factors are concerned.	McVary KT et al., 2001 [49]
8	Cigarette smoking and hormones	Smoking has multiple effects on the secretion and metabolism of hormones.	Marom-Haham L et al., 2016 [51]
9	A systematic review of maternal smoking during pregnancy and fetal measurements with meta-analysis	Maternal smoking during pregnancy is associated with reduced fetal measurements after the first trimester, particularly reduction in the size of head and femur length.	Abraham M et al., 2017 [53]
10	Effects of exposure to smoking on the microbial flora of children and their parents	There are adverse effects of direct and indirect exposure to smoking on colonization with potential pathogens.	Brook I, 2010 [59]
11	Smoking and increased Alzheimer's disease risk: A review of potential mechanisms	The future prevalence of Alzheimer's disease is likely to be reduced with the reduction in the incidence of smoking.	Durazzo TC et al., 2014 [61]
12	Otoacoustic Emissions in Smoking and Nonsmoking Young Adults	In a group of smokers, a general decrease in OAE levels was found.	Jedrzejczak WW et al., 2015 [62]
13	Smoking cessation and response to periodontal treatment	Smoking adversely affects the presence and severity of periodontal diseases and also the outcome of treatment of these diseases.	Alexandridi F et al., 2018 [63]
14	The effects of smoking on fracture healing	Smoking delays the process of fracture healing by mediating via the vasoconstrictive and platelet-activating and aggregating properties of nicotine, the hypoxia-promoting effects of CO and the inhibition of oxidative metabolism by HCN at the cellular level.	Sloan A et al., 2010 [64]
15	Effects of Smoking on Optic Nerve Head Microvasculature Density in Glaucoma	Smoking intensity is associated with the reduction in density of the optic nerve vessel in glaucoma	Eslani M et al., 2022 [67]
16	Effects of chronic smoking on the meibomian glands	Smoking results in meibomian gland damage which may be a risk factor for dry eye.	Muhafiz E et al., 2019 [68]

## Conclusions

Smoking is undisputedly a significant risk factor in the development of many chronic illnesses in humans and thus is a hurdle in living a healthy and happy life. The adverse effects smoking has on the proper functioning of the human body should seldom be neglected. The impact of smoking on the various systems of the human body has been tried to be incorporated in this review article. To completely sum up the effects of smoking in a single article is nearly impossible, as hardly one could find an organ system that is unaffected by smoking. The health benefits that could be achieved due to smoking cessation and the overall well-being it could bring to the community is a highly desirable phenomenon. As smoking is a risk factor in the causation of various diseases, abstinence from smoking provides huge protection from several ailments. The issue of passive smoking also needs to be acknowledged and addressed appropriately to ensure the safety of those who don't smoke. Advanced research, which is currently ongoing, promises to achieve a greater understanding of the various unknown aspects of smoking and hopefully let these studies enlighten more and more smokers and prevent them from dooming their lives with smoke.
